# LROD: An Overlap Detection Algorithm for Long Reads Based on *k*-mer Distribution

**DOI:** 10.3389/fgene.2020.00632

**Published:** 2020-07-29

**Authors:** Junwei Luo, Ranran Chen, Xiaohong Zhang, Yan Wang, Huimin Luo, Chaokun Yan, Zhanqiang Huo

**Affiliations:** ^1^College of Computer Science and Technology, Henan Polytechnic University, Jiaozuo, China; ^2^School of Computer and Information Engineering, Henan University, Kaifeng, China

**Keywords:** overlap detection, alignment, long read, *k*-mer distribution, the third generation sequencing technology

## Abstract

Third-generation sequencing technologies can produce large numbers of long reads, which have been widely used in many fields. When using long reads for genome assembly, overlap detection between any pair of long reads is an important step. However, the sequencing error rate of third-generation sequencing technologies is very high, and obtaining accurate overlap detection results is still a challenging task. In this study, we present a long-read overlap detection (LROD) algorithm that can improve the accuracy of overlap detection results. To detect overlaps between two long reads, LROD first retains only the solid common *k*-mers between them. These *k*-mers can simplify the process of overlap detection. Second, LROD finds a chain (i.e., candidate overlap) that includes the consistent common *k*-mers. In this step, LROD proposes a two-stage strategy to evaluate whether two common *k*-mers are consistent. Finally, LROD uses a novel strategy to determine whether the candidate overlaps are true and to revise them. To verify the performance of LROD, three simulated and three real long-read datasets are used in the experiments. Compared with two other popular methods (MHAP and Minimap2), LROD can achieve good performance in terms of the F1-score, precision and recall. LROD is available from https://github.com/luojunwei/LROD.

## Introduction

Sequencing technologies fragment the genome into a large number of reads, and the process of recombining these reads into a complete DNA sequence is called genome assembly (Nagarajan and Pop, 2013; Ding and Guo, 2018). Read sequencing from next-generation sequencing (NGS) technology (Miller et al., 2010), is usually short, i.e., only a few hundred base pairs in length. Short reads commonly cannot be used to solve problems caused by long repetitive regions (Liao et al., 2020). In addition, NGS polymers commonly lead to some GC bias, which will affect the correctness of the genome assembly (Farrer et al., 2009; Luo et al., 2012). Compared with NGS, third-generation sequencing (TGS) technologies (Schadt et al., 2010), such as single-molecule real-time technology (SMRT) (Levene et al., 2003) and Oxford Nanopore technology (ONT) (Stoddart et al., 2009), can produce longer reads with an average length of 10 kb, with many exceeding 100 kb. This long-read length is sufficient to span most repetitive areas. TGS does not require any polymerase chain reaction process and therefore can avoid GC bias (Ross et al., 2013). It is worth noting that TGS has a higher sequencing random error rate than that of NGS technology. For instance, the sequencing error rate of SMRT is ~15%, and the sequencing error rate of ONT can reach 20%. Fortunately, current studies have shown that high sequencing coverage can correct these random errors. Hence, these long reads are helpful for solving problems caused by sequencing bias and repetitive regions (Ummat and Bashir, 2014; Luo et al., 2016). Currently, many assemblers using long reads have been presented.

Overlap detection is usually the first step of assembly algorithms (Pevzner et al., 2001). For two long reads, the purpose of overlap detection is to determine whether overlap exists. If two long reads have an overlap, overlap detection tools will highlight the two regions from the two long reads separately, which can then be overlapped (Luo et al., 2015a,b).

To identify the overlaps among long reads, two major problems need to be addressed: (1) High sequencing error rate: the long reads from TGS always have high sequencing error rates. Therefore, it is difficult to obtain accurate overlap results. (2) Repetitive regions: long repetitive regions complicate the process of overlap detection. These two problems in the process of overlap detection should attract more attention.

At present, many overlap detection tools that are capable of detecting overlaps among error-prone long reads with different accuracy levels have been developed (Luo et al., 2020).

BLASR (Chaisson and Tesler, 2012) is designed to align long reads to a genome reference and can be used to detect overlaps among long reads. The performance of BLASR depends heavily on the parameter values. For instance, to achieve higher sensitivity, BLASR first needs to know the coverage of the long reads and then chooses the appropriate nBest and nCandidates (*n* is 10 by default) values, which should not be less than the coverage. The running time is also related to the two parameters. BLASR adopts complete aligning and can necessitate more computing resources than are required for downstream processes. DALIGNER (Myers, 2014) is a tool especially designed to detect overlaps among long reads. This method focuses on optimizing the running efficiency in response to the relatively poor running performance of constructing the FM-index suffix array/tree data structure (Ferragina and Manzini, 2005). Its implementation steps are as follows: (a) split long reads into blocks; (b) sort the *k*-mers in each block; and (c) merge the blocks. DALIGNER greatly improves the running efficiency. To increase speed and reduce memory usage, DALIGNER filters out some k-mers. MHAP (Berlin et al., 2015) is a MinHash algorithm (Broder, 1997) that relies on *k*-mer similarity to implement overlap detection for long reads. MHAP first indexes the *k*-mers with multiple hash functions. For all *k*-mers in the two long reads, MHAP builds a sketch list with the minimum value of the hash function and then finds the location of the overlap. Next, MHAP uses the shorter *k*-mers to repeat the previous steps to discover a more accurate overlap. The number of hash functions used by MHAP to build a sketch is always fixed. However, the lengths of the long reads are not equal, and their sensitivity is affected when the length of the reads varies widely. For shorter reads, MHAP inevitably wastes some memory. For longer reads, accurate overlap detection is difficult to achieve because too few *k*-mers exist. Minimap2 (Li, 2016, 2018) is an overlap detection tool, that applies the idea of the sketch from MHAP but uses minimizers as a simplified representation instead. Similar to MHAP, Minimap2 saves *k*-mers in a hash table. In addition, Minimap2 adopts a sorting strategy inspired by DALIGNER to improve the running efficiency.

In this paper, we develop an approach named long-read overlap detection (LROD) to detect overlaps among long reads based on the *k*-mer distribution. The main contributions of LROD are the following. To address the problem caused by sequencing errors and repetitive regions, for two long reads, LROD first finds all solid common *k*-mers between them. Solid *k*-mers might not include sequencing errors and may come from repetitive regions, which helps to simplify the process of overlap detection. Then, LROD employs a two-stage strategy to determine whether two common *k*-mers are consistent, i.e., whether the middle regions between them overlap. Then, LROD finds a chain that comprises consistent common *k*-mers, which indicates a candidate overlap. Finally, LROD revises the candidate overlap and utilizes a new evaluation method to determine whether the candidate overlap is true. The experimental results demonstrate that LROD performs better than MHAP and Minimap2 in terms of the F1-score.

## Materials and Methods

In this paper, LROD is used to detect the overlaps among long reads based on the distribution of *k*-mers. One *k*-mer is a substring with a length *k* in long reads. Suppose that the length of a long read is *L*; then, the number of *k*-mers in the long read is (*L – k* + *1*). LROD first identifies solid *k*-mers and keeps them, removing all other *k*-mers. Then, LROD constructs a *k*-mer hash table. For two long reads, LROD uses Algorithm 1 to detect any overlap between them. As shown in Algorithm 1, LROD first finds the common *k*-mer set (CKS) between two long reads *R*_1_ and *R*_2_. Second, based on the CKS, LROD attempts to search a chain for consistent common *k*-mers, which correspond to a candidate overlap. Third, LROD further evaluates the candidate and determines the final overlap. In the following sections, we describe each step in detail.

**Algorithm 1:**
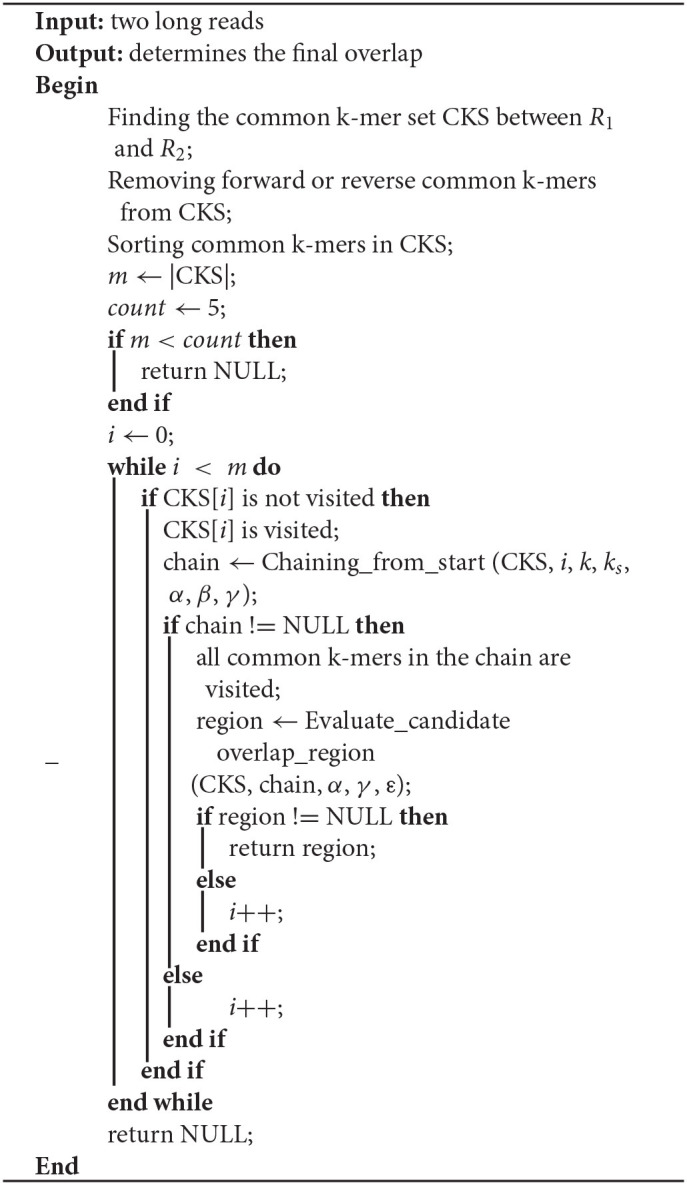
Finding_overlap_region (*R*_1_, *R*_2_, *k*, *k*_*s*_, α, β, γ, ε)

### Selecting Solid *k*-mers

For two long reads, LROD utilizes the common *k*-mers between them to determine whether they overlap. However, the high sequencing error rate of TGS usually leads to negative common *k*-mers, and repetitive regions can cause a position contradiction among common *k*-mers. For a long-read dataset, one *k*-mer with a small frequency commonly includes sequencing errors, whereas, one *k*-mer with a large frequency usually originates from a repetitive region (Liu et al., 2013). Hence, LROD selects only *k*-mers whose frequencies are in the interval [*f*_min_, *f*_max_] as solid *k*-mers, where *f*_min_ and *f*_max_ are two thresholds that are calculated by LROD. Using only solid *k*-mers allows LROD to avoid some problems caused by sequencing errors and repetitive regions.

Before calculating the values of *f*_min_ and *f*_max_, LROD should determine the value of *k*. A larger value of *k* helps to resolve repetition-related problems, but decreases the number of common *k*-mers between two long reads. A smaller value of *k* will introduce more negative common *k*-mers, and complicate the process of overlap detection. LROD sets *k* to 15 by default. Next, LROD uses the method described below to select solid *k*-mers.

For a long-read dataset, LROD first uses DSK (Rizk et al., 2013), a *k*-mer counting program, to calculate the frequency of each *k*-mer in the dataset. If the frequency of a *k*-mer is one, then only one read contains this *k*-mer, which means that it is useless for finding any overlap between two long reads. For LROD, the minimum frequency of the *k*-mer is 2, that is, *f*_min_ = 2 by default. This threshold can filter out a large number of *k*-mers that might include sequencing errors.

The value of *f*_max_ should be determined based on the coverage of the long-read set and the characteristics of the genome. If *f*_max_ is high, some *k*-mers from repetitive regions may be kept in the following steps. If *f*_max_ is low, some *k*-mers that do not originate from repetitive regions may be ignored. LROD develops a method to calculate *f*_max_ based on the frequency of *k*-mers. *F*(*x*) refers to the number of *k*-mers whose frequency is *x, x* = 1, 2, 3…, *h*, where *h* is the maximum *k*-mer frequency. For example, a *k*-mer set {AAT, ATA, TAG, TAG, AGT, ATA, AAT, AGT, AGT} exists. For this *k*-mer set, no *k*-mer appears once, and thus, *F*(1) = 0. *F*(2) = 3, which means that three *k*-mers appear twice, i.e., “AAT, ATA, TAG.” *F*(3) = 1, which means that only one *k*-mer, i.e., “AGT,” is repeated three times.

Then, *S*(*y*) is used to calculate the cumulative sum of *F*(*x*), as described in equation 1. When *f* is the smallest value such that *S*(*f*) > θ^*^S(*h*), we set *f*_max_ = *f*, and θ = 0.9 by default, and *S*(*h*) is the total frequency of *k*-mers whose frequencies are not smaller than 2.

(1) $$\begin{array}{l} S\left(y\right)=\overset{y}{\underset{x=f_{\text{min}}}{\sum }}F\left(x\right) \end{array}$$

After determining the interval [*f*_min_, *f*_max_], the *k*-mers whose frequencies are not in this interval are ignored in subsequent steps. Overlap detection using only solid *k*-mers can minimize the impacts of sequencing errors and repetitive regions, and improve the accuracy of results.

The remaining solid *k*-mers are indexed by using a *k*-mer hash table with the *k*-mers as keys. For a specific *k*-mer, the *k*-mer hash table enables LROD to quickly identify the long reads that include it. At the same time, LROD can find locations and orientations in these long reads. As a result, LROD can quickly find a CKS for two long reads based on the *k*-mer hash table.

### Detecting Overlap Between Two Long Reads

LROD selects two long reads *R*_1_ and *R*_2_ to detect whether they overlap. If they overlap, LROD will give the region in *R*_1_ that overlaps with another region in *R*_2_. The process of overlap detection for *R*_1_ and *R*_2_ is described below.

#### Finding a Common k-mer Set Between R_1_ and R_2_

First, LROD extracts *k*-mers from *R*_1_ with a step (*s*, 1 in default), and selects *k*-mers which appear in *R*_2_ through the *k*-mer hash table. Then, LROD gets a common *k*-mer set (CKS). If one *k*-mer in a long read appears twice or more in another long read, LROD deletes it from CKS. The *i*-th common *k*-mer in CKS is represented by a four-tuples (*P*_1*i*_, *O*_1*i*_, *P*_2*i*_, *O*_2*i*_). *P*_1*i*_ and *P*_2*i*_ are the starting positions of the common *k*-mer in *R*_1_ and *R*_2_ respectively. *O*_1*i*_ and *O*_2*i*_ are the orientations of the common *k*-mer in *R*_1_ and *R*_2_, respectively. If the *i*-th common *k*-mer has the same orientation (*O*_1*i*_ = *O*_2*i*_), the common *k*-mer is a positive common *k*-mer, otherwise, it is an opposite common *k*-mer. LROD uses *M* to represent the number of positive common *k*-mers, and *N* to indicate the number of opposite common *k*-mers. If *M* > *N* and *M* > *count* (*count* = 5), LROD keeps the positive common *k*-mers and ignores the opposite common *k*-mers. If *N* > *M* and *N* > *count*, LROD keeps the opposite *k*-mers and ignores the positive *k*-mers. The remaining common *k*-mers in the CKS are sorted in ascending order based on their positions in *R*_1_. If *R*_1_ and *R*_2_ do not satisfy any of the above two conditions, LROD concludes that they do not have an overlap and processes another pair of long reads.

#### Chaining

In this step, LROD aims to find a chain from the CKS that consists of some consistent common *k*-mers, and corresponds to a candidate overlap. Algorithm 2 shows the pseudocode of chaining. First, the starting common *k*-mer is added to the chain. Then, LROD searches for the first subsequent common *k*-mer, which is consistent with the previous common *k*-mer in the chain. When one consistent common *k*-mer is found, it is appended to the chain. LROD repeats this process until all common *k*-mers are visited. Finally, LROD obtains a chain.

**Algorithm 2:**
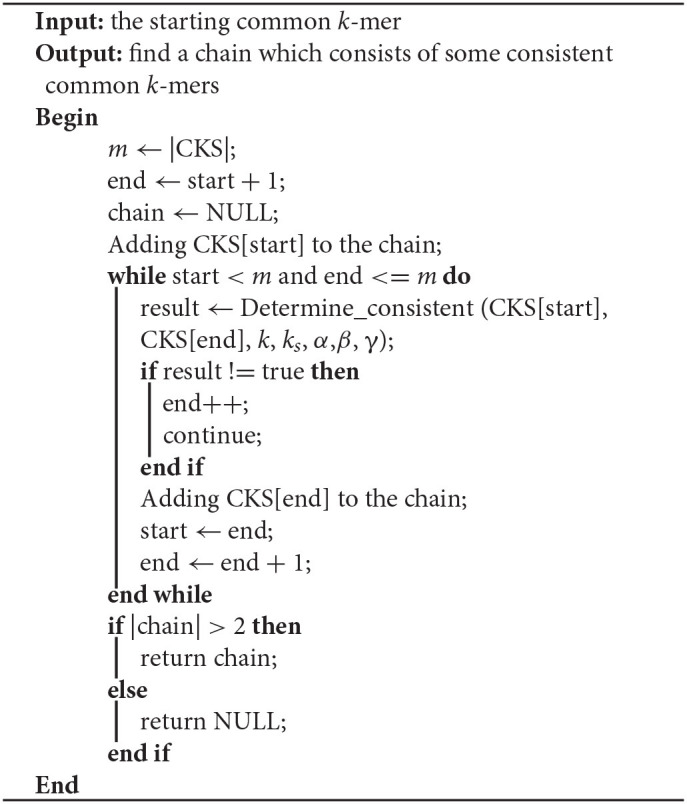
Chaining_from_start (CKS, start, *k*, *k*_*s*_, α,β, γ)

**Figure 1:**
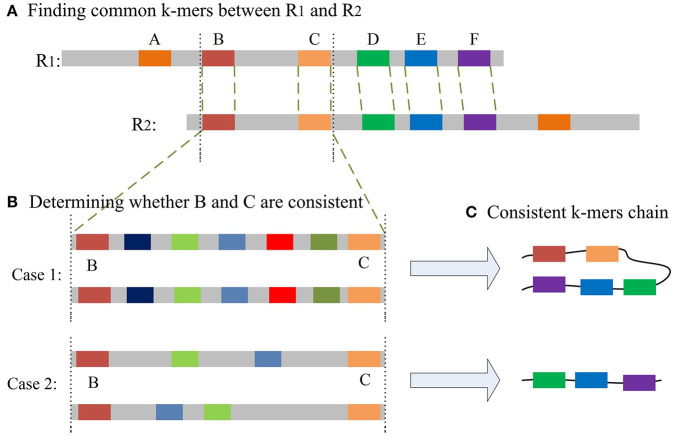
**(A)** A–F are the common *k*-mers in the two long reads, D–F is the common consistent *k*-mer that can be judged in the first stage. The distance between B and C is slightly longer, but less than β. **(B)** Aligning sequences between B and C with smaller *k*_*s*_-mers in the second stage may result in two situations. Case 1: Multiple *k*_*s*_-mers can be compared between B and C; Case2: there are no common consistent *k*_*s*_-mers between B and C. **(C)** According to different situations, two different consistent chains will be generated.

The most important issue in finding a chain is how to decide whether two common *k*-mers are consistent. For two common *k*-mers, their distances in the two long reads can be calculated. When the two distances are large or differ too much, the two common *k*-mers might be inconsistent. LROD employs a two-stage strategy for this issue, an example of which is shown in Figure 1. Algorithms 3–5 show the pseudocode for determining whether two common *k*-mers are consistent. In the first stage as shown in Algorithm 4, LROD presents some conditions to evaluate. If they cannot be determined in the first stage, LROD uses the second stage as shown in Algorithm 5 to further analyse whether they are consistent based on smaller *k*_*s*_-mers (*k*_*s*_ < *k*).

**Algorithm 3:**
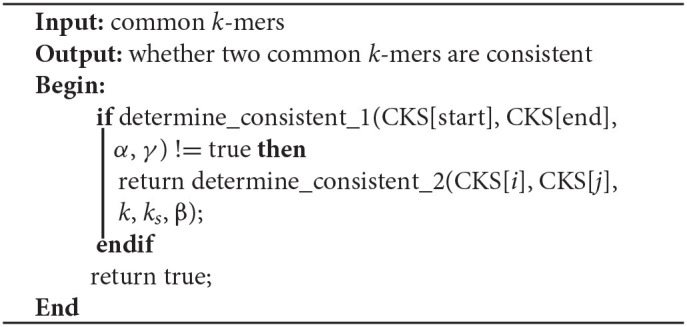
Determine_consistent (CKS[start], CKS[end], *k*, *k*_*s*_, α,β,γ)

**Algorithm 4:**
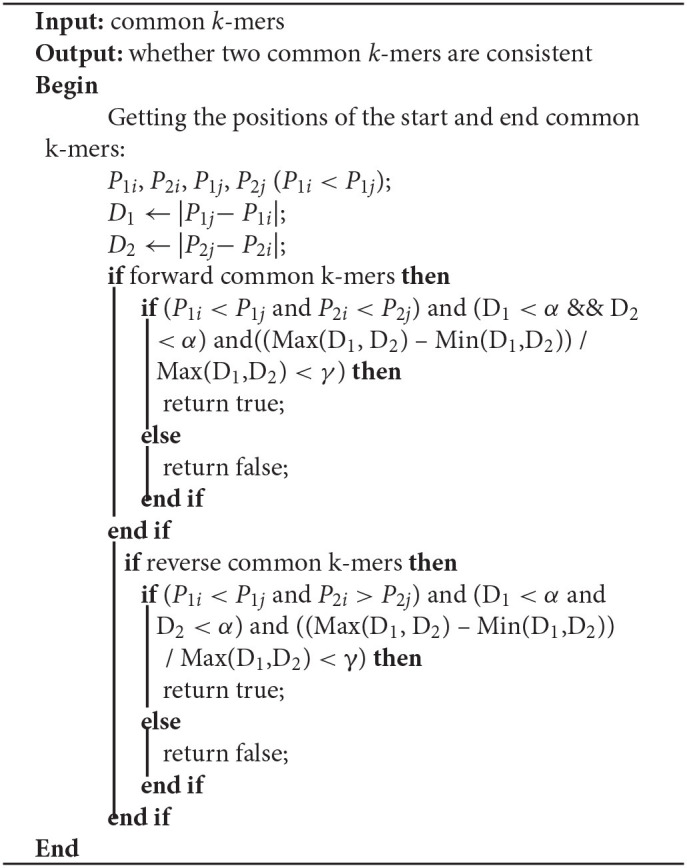
Determine_consistent_1 (CKS[start], CKS[end], *k*, *k*_*s*_, α,γ)

**Algorithm 5:**
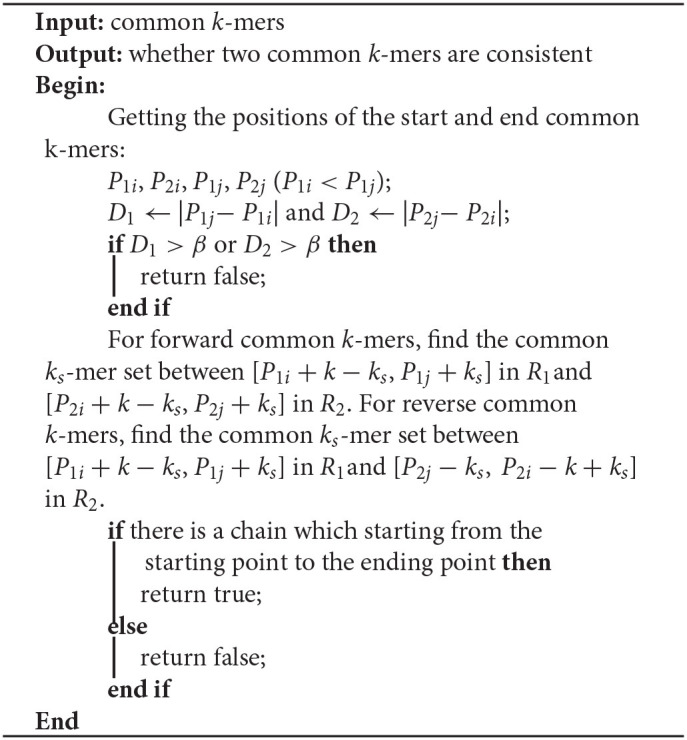
Determine_consistent _2 (CKS[start], CKS[end], *k*, *k*_*s*_, β)

**Algorithm 6:**
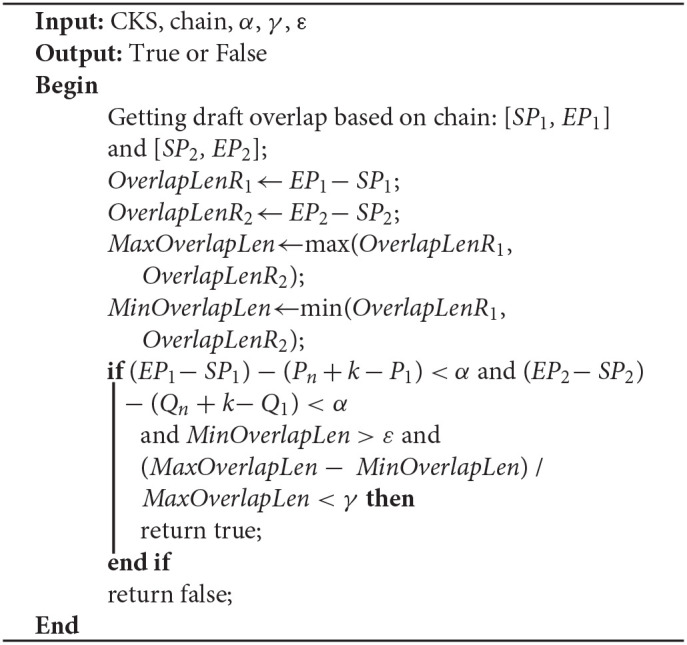
Evaluate_candidate_overlap_region(CKS, chain, α, γ, ε)

For two common *k*-mers (*P*_1*i*_, *O*_1*i*_, *P*_2*i*_, *O*_2*i*_) and (*P*_1*j*_, *O*_1*j*_, *P*_2*j*_, *O*_2*j*_), two distances *D*_1_ = |*P*_1*j*_ − *P*_1*i*_| and *D*_2_ = |*P*_2*j*_ − *P*_2*i*_| can be calculated. In Algorithm 4, LROD uses C1, C2, C3, and C4 to evaluate their consistency. The four conditions are listed below. C1 means that they are forward, while C2 means the reverse. Due to the high sequencing error rate of TGS, we should allow a distance between two consecutive consistent common *k*-mers. However, the larger the distance is, the greater the number of sequencing errors that exist. Hence, C3 specifies the distance threshold α between two consistent common *k*-mers. However, α is difficult to determine. A small α will miss some consistent common *k*-mers, and a large α will accept more inconsistent common *k*-mers. In this stage, LROD adopts a small value of α (400 by default) to select consistent common *k*-mers with high confidence. C4 specifies the maximum difference between *D*_1_ and *D*_2_ (γ =0.3). When the two common *k*-mers satisfy the conditions, LROD considers them to be consistent.

C1: *P*_1*i*_ < *P*_1*j*_ and *P*_2*i*_ < *P*_2*j*_;

C2: *P*_1*i*_ < *P*_1*j*_ and *P*_2*i*_ > *P*_2*j*_;

C3: D_1_ < α and D_2_ < α;

C4: (Max(D_1_, D_2_) – Min(D_1_,D_2_)) / Max(D_1_,D_2_) < γ

If Algorithm 4 returns false, LROD will utilize Algorithm 5 to further evaluate the consistency between the two common *k*-mers. In Algorithm 5, LROD adopts a large β (1,500 by default), which provides more candidate common *k*-mers. To identify correct consistent common *k*-mers, LROD finds small *k*_*s*_-mers (*k*_*s*_ < *k*) from two regions in *R*_1_ and *R*_2_ between these two common *k*-mers. If two common *k*_*s*_-mers satisfy C3 and C4, they will be linked. If LROD can find a path from the starting common *k*_*s*_-mer to the ending common *k*_*s*_-mer, then Algorithm 5 returns true.

After obtaining the chain, the number of common *k*-mers in the chain should be larger than 2. Finally, if the common *k*-mers in the chain are positive, LROD concludes that *R*_1_ and *R*_2_ possibly come from the same strand. Otherwise, they might come from reverse strands. Moreover, LROD obtains two draft overlaps [*P*_1_, *P*_*n*_ + *k*] and [*Q*_1_, *Q*_*n*_ + *k*] on *R*_1_ and *R*_2_, respectively, where *P*_1_ and *Q*_1_ are the first *k*-mers in the chain, and *P*_*n*_ and *P*_*n*_ are the last *k*-mers in the chain.

#### Determining the Final Overlap

**Figure 2:**
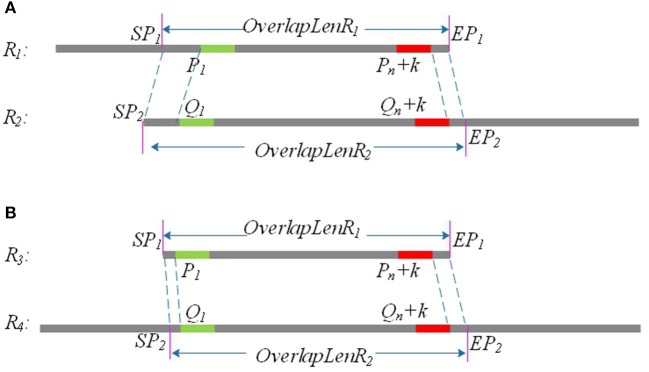
*P*_1_ and *Q*_1_ are the start positions of the common positive *k*-mer on *R*_1_ and *R*_2_, and (*P*_*n*_ + *k*) and (*Q*_*n*_ + *k*) are the end positions of the common positive *k*-mer on *R*_1_ and *R*_2_. *SP*_1_ and *EP*_1_ are the start position and end position of the final overlap on *R*_1_, respectively, and *SP*_2_ and *EP*_2_ are the start position and end position of the final overlap on *R*_2_. **(A)** Partial overlap: The right end of one long read aligning with the left end of the other long read. **(B)** Full overlap: A long read is completely aligned to a part of the other long read.

Due to sequencing errors, the above candidate overlap may deviate somewhat from the real overlap. Suppose the true overlap on *R*_1_ is [*SP*_1_, *EP*_1_], and the true overlap on *R*_2_ is [*SP*_2_, *EP*_2_]. The lengths of *R*_1_ and *R*_2_ are *Len*_1_ and *Len*_2_, respectively. LROD uses the following method to revise the candidate overlap and obtain the true overlap for *R*_1_ and *R*_2_. An example is shown in Figure 2.

1. If *P*_1_ > *Q*_1_ and *Len*_1_ − *P*_*n*_ <= *Len*_2_ − *Q*_*n*_, *SP*_1_ = *P*_1_ − *Q*_1_, *EP*_1_ = *Len*_1_; *SP*_2_ = 1, *EP*_2_ = *Q*_*n*_ + *Len*_1_ − *P*_*n*+1_, as shown in Figure 1A.
2. If *P*_1_ < *Q*_1_ and *Len*_1_ − *P*_*n*_ <= *Len*_2_ − *Q*_*n*_, *SP*_1_ = 1, *EP*_1_ = *Len*_1_; *SP*_2_ = *Q*_1_ − *P*_1_, *EP*_2_ = *Q*_*n*_ + *Len*_1_ − *P*_*n*+1_, as shown in Figure 1B.
3. If *P*_1_ > *Q*_1_and *Len*_1_ − *P*_*n*_ > *Len*_2_ − *Q*_*n*_, *SP*_1_ = *P*_1_ − *Q*_1_, *EP*_1_ = *P*_*n*_ + *Len*_2_ − *Q*_*n*_; *SP*_2_ = 1, *EP*_2_ = *Len*_2_.
4. If *P*_1_ < *Q*_1_ and *Len*_1_ − *P*_*n*_ > *Len*_2_ − *Q*_*n*_, *SP*_1_ = 1, *EP*_1_ = *P*_*n*_ + *Len*_2_ − *Q*_*n*_; *SP*_2_ = *Q*_1_ − *P*_1_, *EP*_2_ = *Len*_2_.

After the above processing, the real overlap on *R*_1_ and *R*_2_ can be obtained.

As shown in Figure 2, the length of the overlap on *R*_1_ is *OverlapLenR*_1_ = *EP*_1_ − *SP*_1_, and the length of the overlap on *R*_2_ is *OverlapLenR*_2_ = *EP*_2_ − *SP*_2_. We use *MaxOverlapLen* and *MinOverlapLen* to represent the maximum overlap length and the minimum overlap length, respectively. *MaxOverlapLen* = max(*OverlapLenR*_1_, *OverlapLenR*_2_), and *MinOverlapLen* = min(*OverlapLenR*_1_, *OverlapLenR*_2_).

When *R*_1_ and *R*_2_ satisfy the following three conditions, LROD considers *R*_1_ and *R*_2_ to have an overlap. The overlaps are [*SP*_1_, *EP*_1_] and [*SP*_2_, *EP*_2_] on *R*_1_ and *R*_2_, respectively. Otherwise, no overlap exists between them. ε is the threshold of the overlap length (500 by default).

1. (*EP*_1_ − *SP*_1_) − (*P*_*n*_ + *k* − *P*_1_) < α and (*EP*_2_ − *SP*_2_) − (*Q*_*n*_ + *k* − *Q*_1_) < α;
2. *MinOverlapLen* > ε;
3. (*MaxOverlapLen* − *MinOverlapLen*) / *MaxOverlapLen* < γ.

### Parameters of LROD

LROD has nine tunable parameters that can affect the final experimental results. These parameters can be categorized into three groups. The first group contains five parameters, namely, *f*_min_, θ, *s, k*, and *k*_s_, which are used to determine the common *k*-mers between two long reads. More solid *k*-mers can be retained by increasing the value of *f*_min_ and reducing the value of θ, though doing so will also increase the number of false solid *k*-mers. We assign 1 as the default value for parameter *s*, which can retain all solid *k*-mers and find all common *k*-mers between two long reads. Generally, the detection of overlaps based on short *k*-mers (*k* = 9) is sensitive, while Minimap2 sets *k* = 15 to detect overlaps. Therefore, we set *k* = 15 and *k*_*s*_ = 9 by default to balance the sensitivity and specificity. Specifically, the memory requirement and running time of LROD decrease when the values of these parameters increase. The second group consists of three parameters, represented as α, β, and γ, which are utilized to evaluate whether two common *k*-mers are consistent. Minimap2 uses a 500 bp band-width to find collinear minimizers. Here, we set the default α to 400 to select consistent common *k*-mers with high confidence. Moreover, we use β = 1,500 and *k*_*s*_-mers (*k*_*s*_ < *k*) to further determine whether they are consistent. Essentially, if two common *k*-mers are consistent, the two distances between them should be similar. Thus, when the difference between the two distances is larger than γ (0.3 by default), they are regarded as inconsistent by LROD. The values of these parameters will need to be changed according to the sequencing error rate of TGS. If the error rate is low, reducing the values of these parameters could improve the precision of the result. The last group contains parameter ε. LROD outputs only the overlaps whose lengths are larger than ε.

To further examine the impact of these parameters on the results of overlap detection, we conducted LROD with different values of four parameters: α, β, γ, and θ. The results are shown in the Supplementary Material.

## Results and Discussion

To verify the effectiveness of the proposed method in this paper, we used three simulated and three real datasets to benchmark LROD, MHAP and Minimap2; the performance was verified with *k*-mer lengths of *k* = 13 and *k* = 15, respectively. The three real datasets are from genomes of *Escherichia coli* (*E. coli), Caenorhabditis elegans* (*C. elegans*), and *humans*, which were sequenced by SMRTs. The real datasets related to *E. coli* and *C. elegans* are available from schatzlab.cshl.edu/data/ectools/. The real human dataset is NA20300 (SRR9683669). The three real datasets are herein referred to as *E. coli_Real, C. elegans_Real*, and *Human_Real*. In this paper, we used SURVIVOR (Jeffares et al., 2017) to obtain three simulated datasets: 10X coverage *E. coli* (*E. coli-10*), 20X coverage *E. coli* (*E. coli-20*), and 10X coverage human chromosome 20 (*chr20-10*). For these long-read datasets, we retained the long reads whose lengths were longer than 2,000 bp in the following experiments. Table 1 shows the details of the long-read datasets, including the genomic length, average length of reads, number of reads, and coverage.

**Table 1:** Details of datasets.

**Datasets**	**Genomic length (Mbp)**	**Average length of reads(bp)**	**Number of read**	**Coverage**
*E. coli-10*	~4.6	6,555	6,955	~10
*E. coli-20*	~4.6	6,619	13,911	~20
*chr20-10*	~6.4	6,621	96,574	~10
*E. coli_Real*	~4.6	4,185	6,972	~7
*C. elegans_Real*	~99.9	4,091	188,559	~77
*Human_Real*	~3,157	25,890	461,247	~3.78

For the three simulated datasets, we can directly obtain the real overlaps among the long reads. For the three real datasets, we used BLASR to align these long reads against the reference genomes. We retained only those reads whose aligning quality was >85%, and the long reads were completely aligned on the genome reference. Then, we were able to acquire real overlaps among these long reads based on their alignment positions. The obtained overlaps were used to evaluate the performance of the overlap detection tools. All tools were run with 10 threads on a computer with 128 GB of memory. During the experiments, the wall time of LROD can be reduced by adopting a larger thread number.

### Results

For the real human dataset, when *k* = 13, Minimap2 and LROD did not end with 10 threads after 10 days, and the memory requirement of MHAP was larger than the memory capacity of our computer (128 GB). Therefore, we do not give the results for the human dataset with *k* = 13.

**Figure 3:**
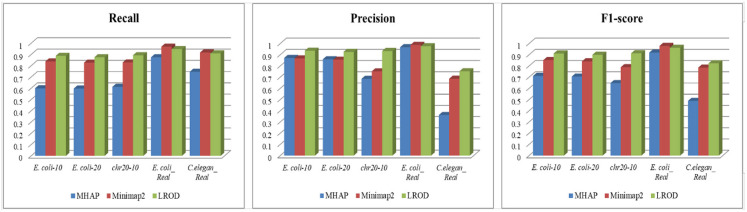
Overlap detection with *k* = 13.

**Figure 4:**
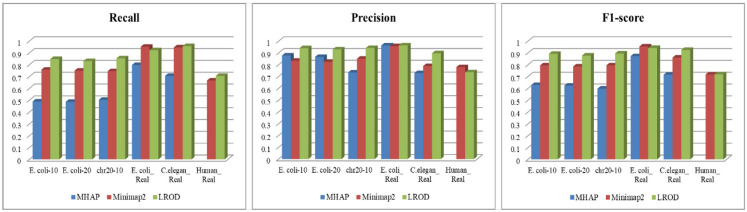
Overlap detection with *k* = 15.

As shown in Table 2 and Figure 3, LROD obtained satisfactory results for these datasets. Especially in the simulated datasets, for most cases, the precision, recall, and F1-score of LROD were higher than those of Minimap2 and MHAP. Although LROD was slightly inferior to Minimap2 in terms of the F1-score for *E. coli_Real*, it also achieved a similar performance to that of Minimap2. When *k* = 15, as shown in Table 2 and Figure 4, LROD was superior to the other two tools on the basis of the F1-score.

**Table 2:** Overlap detection on the datasets.

***k***	**Dataset**	**Precision**			**Recall**			**F1-score**		
		**MHAP**	**Minimap2**	**LROD**	**MHAP**	**Minimap2**	**LROD**	**MHAP**	**Minimap2**	**LROD**
*k* = 13	*E. coli*-*10*	0.871	0.866	0.935	0.599	0.837	0.887	0.710	0.851	0.910
	*E. coli*-*20*	0.859	0.855	0.924	0.597	0.827	0.875	0.704	0.841	0.899
	*chr20-10*	0.685	0.752	0.933	0.612	0.829	0.893	0.646	0.788	0.912
	*E. coli_Real*	0.967	0.987	0.976	0.875	0.969	0.948	0.919	0.978	0.962
	*C. elegan*_*Real*	0.362	0.685	0.752	0.746	0.917	0.909	0.487	0.785	0.824
*k* = 15	*E. coli-10*	0.878	0.834	0.941	0.490	0.759	0.849	0.629	0.795	0.893
	*E. coli-20*	0.866	0.825	0.930	0.487	0.751	0.831	0.624	0.786	0.878
	*chr20-10*	0.734	0.851	0.942	0.504	0.746	0.855	0.598	0.795	0.896
	*E. coli*_*Real*	0.963	0.957	0.964	0.798	0.953	0.924	0.873	0.955	0.943
	*C. elegan*_*Real*	0.729	0.789	0.897	0.706	0.947	0.958	0.717	0.861	0.926
	*Human_Real*	–	0.779	0.736	–	0.667	0.706	–	0.719	0.720

#### Simulated Datasets

For *E. coli-10, E. coli-20*, and *chr20-10*, LROD consistently had the best results in terms of the precision, recall, and F1-score. For these three datasets, the precision of LROD was consistently above 90%. Although the recall of LROD did not reach 90%, all values were >85% and higher than those of the other two tools. The average F1-scores for LROD were 5% and 20% higher than those of Minimap2 and MHAP, respectively. The precision of LROD exceeded 92%, and the recall of LROD exceeded 83%. Both the precision and recall of LROD were higher than those of Minimap2 in both the precision and recall. Therefore, the F1-score of LROD ranked first for these datasets.

#### Real Datasets

As shown in Table 2 and Figures 3, 4, for *E. coli_Real*, the F1-scores of LROD and Minimap2 were >95%. Although LROD did not obtain the highest F1-score, it was very close to that of Minimap2. For *C. elegans_Real*, the precisions of Minimap2 and MHAP were smaller than that of LROD. Both Minimap2 and LROD showed good recall, and LROD obtained the best F1-score. For *Human_Real*, the F1-scores of Minimap2 and LROD were similar. Note that the memory requirement of MHAP exceeded the memory capacity of our computer; hence, we did not give its result.

#### Running Time and Memory Requirements

We compared the computational requirements among the three tools for the six datasets. The results are shown in Table 3. The memory consumption of MHAP is very large, and Minimap2 has the lowest memory consumption. Although the memory consumption of LROD is greater than that of Minimap2, it is smaller than that of MHAP. In terms of running time (CPU time), LROD is better than MHAP. The surprising running time and memory consumption of Minimap2 have attracted attention from many researchers. Note that all tools can use more threads to reduce the wall time.

**Table 3:** Running time and memory.

***k***	**Dataset**	**MHAP**		**Minimap2**		**LROD**	
		**Running time**	**Memory (Mb)**	**Running time**	**Memory (Mb)**	**Running time**	**Memory (Mb)**
*k* = 13	*E. coli-10*	4 m 29 s	39,703	0 m 45 s	1,251	2 m 4 s	1,491
	*E. coli-20*	9 m 15 s	39,971	2 m 30 s	1,612	5 m 5 s	2,336
	*chr20-10*	87 m 44 s	42,748	125 m 26 s	8,441	59 m 1 s	11,487
	*E. coli_Real*	4 m 14 s	39,501	0 m 26 s	1,129	1 m 36 s	1,238
	*C. elegans_Real*	28,813 m 15 s	42,156	1,753 m 38 s	25,940	14,625 m 23 s	36,708
*k* = 15	*E. coli-10*	6 m 3 s	41,163	0 m 17 s	2,644	2 m 14 s	3,326
	*E. coli-20*	12 m 34 s	42,959	0 m 48 s	2,972	5 m 51 s	4,248
	*chr20-10*	88 m 18 s	43,641	21 m 52 s	7,625	46 m 38 s	11,906
	*E. coli_Real*	4 m 42 s	41,099	0 m 1 s	2,513	1 m 40 s	3,036
	*C. elegans_Real*	377 m 17 s	44,414	292 m 45 s	15,522	620 m 16 s	17,418
	*Human_Real*	–	–	424 m 42 s	35,814	4,402 m 10 s	51,906

### Discussion

MHAP is a MinHash algorithm used to implement overlap detection based on *k*-mer similarity. For two long reads, MHAP builds a fixed number of *k*-mer sketch lists with the minimum value of the hash functions and then finds the location of the overlap. Only a certain number of *k*-mers are left for each read because the length of each read is different; the shorter the reads are, the more accurate the MHAP detection. Minimap2 ingeniously combines the advantages of its predecessor's algorithms, including DALIGNER, MHAP, and GraphMap (Sović et al., 2016). Minimap2 adopts the streaming SIMD extension instruction calculation method. Hence, the efficiency of Minimap2 is very high. LROD is an overlap detection algorithm based on the *k*-mer distribution. It starts with solid *k*-mers selected based on the frequency distribution of *k*-mers for the entire long-read dataset. Although LROD performs well-according to the experimental results, it has an obvious shortcoming in terms of running time. In the future, we will focus on improving the LROD calculation performance module, increasing the calculation speed, and reducing the running time.

## Conclusion

In this paper, we develop an overlap detection tool named LROD, which performs well in detecting overlaps among the long reads obtained from TGS technology. LROD first selects solid *k*-mers, which can reduce the computation time and memory requirements and can avoid some problems caused by sequencing errors and repetitive regions. For two long reads, LROD first finds their common *k*-mers. Second, LROD adopts a two-stage strategy to detect whether two common *k*-mers are consistent and to search for a chain that corresponds to a candidate overlap. Finally, LROD further evaluates the candidate overlap and determines the real overlap between the two long reads. The experimental results on three simulated datasets and three real datasets show that LROD can obtain satisfactory overlap detection results in terms of the precision, recall, and F1-score.

## Data Availability Statement

Publicly available datasets were analyzed in this study. This data can be found at Schatz Lab (http://schatzlab.cshl.edu/data/ectools/; http://schatzlab.cshl.edu/data/nanocorr/). All source codes for LROD are available at Github (https://github.com/luojunwei/LROD).

## Author Contributions

JL, ZH, and RC proposed the method and designed the experiments. JL and RC wrote the paper. YW and XZ provided guidance for this paper. HL and CY provided support for the completion of the experiment. All authors contributed to the article and approved the submitted version.

## Conflict of Interest

The authors declare that the research was conducted in the absence of any commercial or financial relationships that could be construed as a potential conflict of interest.

## Abbreviations

A: adenine
T: thymine
C: cytosine
G: guanine
NGS: next generation sequencing
PCR: polymerase chain re-action
SMRT: single molecule sequencing
PB: pacific biosciences
SMRT: single molecule real time
ONT: oxford nanopore technology
OLC: overlap-layout-consensus
MHAP: MinHash alignment process
BLAST: basic local alignment search tool
BLAT: the BLAST like alignment tool
BLASR: basic local alignment with successive refinement
SSE: streaming SIMD extensions.
