# Supplementary material for: LROD: An Overlap Detection Algorithm for Long Reads Based on k-mer Distribution
Source: Front Genet. 2020 Jul 29;11:632. doi: 10.3389/fgene.2020.00632 (PMC7403501; doi:10.3389/fgene.2020.00632)
Supplement: Supplementary file 1 [file Data_Sheet_1.PDF]

Supplementary Material

For further examining the impact of these parameters on the results of overlap detection, we conduct LROD with different values of four parameters: α (400 in default), β (1500 in default), γ (0.3 in default) and θ (0.9 in default). The results are shown in Table S1-S4. In Table S1, we set α be 300, 400, 500, 700, other parameters in default. In Table S2, we set β be 1000, 1500, 2000, other parameters in default. In Table S3, we set θ be 0.7, 0.8, 0.9, 0.95, other parameters in default. In Table S4, we set γ be 0.2, 0.3, 0.4, other parameters in default. From Table S1-S4, we can see that different values of β and θ influence the final results in terms of F1-score. For small genome data, LROD can get a better result with large values of β and θ. And for large genome data, LROD can get a better result with small values of β and θ. The two parameters α and γ have little effects on the final result.

Table S1. Results with different values of α

Dataset		α= 300	α= 400	α= 500	α= 700
E. coli-10	Precision	0.941128	0.941068	0.940944	0.940947
	Recall	0.849611	0.849611	0.849553	0.849591
	F1-score	0.893031	0.893004	0.892916	0.892939
E. coli-20	Precision	0.930230	0.930198	0.930193	0.930174
	Recall	0.831438	0.831457	0.831462	0.831471
	F1-score	0.878064	0.878060	0.878061	0.878057
chr20-10	Precision	0.942114	0.942115	0.942093	0.942083
	Recall	0.855165	0.855177	0.855152	0.855153
	F1-score	0.896537	0.896544	0.896519	0.896516
E. coli	Precision	0.964815	0.964355	0.963928	0.963344
	Recall	0.921202	0.924131	0.926443	0.927356
	F1-score	0.942504	0.943815	0.944814	0.945007
C. elegans	Precision	0.897772	0.897060	0.896328	0.894254
	Recall	0.957469	0.958047	0.958704	0.959627
	F1-score	0.926660	0.926551	0.926467	0.925788
human	Precision	0.736960	0.736254	0.735154	0.731806
	Recall	0.703899	0.706253	0.708160	0.709412
	F1-score	0.720050	0.720942	0.721405	0.720435

Table S2. Results with different values of β

Dataset		**β** = 1000	**β** = 1500	**β** = 2000
E. coli-10	Precision	0.942963	0.941068	0.939045
	Recall	0.826345	0.849611	0.853638
	F1-score	0.880811	0.893004	0.894307
E. coli-20	Precision	0.931665	0.930198	0.928903
	Recall	0.805949	0.831457	0.836356
	F1-score	0.864259	0.878060	0.880204
chr20-10	Precision	0.942195	0.942115	0.941964
	Recall	0.840793	0.855177	0.855759
	F1-score	0.888610	0.896544	0.896795
E. coli	Precision	0.967332	0.964355	0.962510
	Recall	0.908812	0.924131	0.926658
	F1-score	0.937160	0.943815	0.944244
C. elegans	Precision	0.932931	0.897060	0.861098
	Recall	0.929111	0.958047	0.961918
	F1-score	0.931017	0.926551	0.908720
human	Precision	0.854975	0.736254	0.628919
	Recall	0.657835	0.706253	0.476657
	F1-score	0.743560	0.720942	0.542303

Table S3. Results with different values of θ

Dataset		$\theta = 0.7$	$\theta = 0.8$	$\theta = 0.9$	$\theta = 0.95$
E. coli-10	Precision	0.942400	0.942162	0.941068	0.939624
	Recall	0.758468	0.829510	0.849611	0.856381
	F1-score	0.840489	0.882255	0.893004	0.896074
E. coli-20	Precision	0.929970	0.930067	0.930198	0.929782
	Recall	0.646394	0.764631	0.831457	0.839762
	F1-score	0.762675	0.839274	0.878060	0.882482
chr20-10	Precision	0.941160	0.941623	0.942115	0.942016
	Recall	0.787239	0.822764	0.855177	0.862077
	F1-score	0.857346	0.878190	0.896544	0.900275
E. coli	Precision	0.975675	0.969342	0.964355	0.960933
	Recall	0.860222	0.910076	0.924131	0.930769
	F1-score	0.914318	0.938774	0.943815	0.945611
C. elegans	Precision	0.968632	0.950934	0.897060	0.848182
	Recall	0.925842	0.945711	0.958047	0.961712
	F1-score	0.946754	0.948315	0.926551	0.901386
human	Precision	0.939383	0.919426	0.736254	0.199290
	Recall	0.662103	0.685430	0.706253	0.716512
	F1-score	0.776739	0.785369	0.720942	0.311844

Table S4. Results with different values of γ

Dataset		γ = 0.2	γ = 0.3	γ = 0.4
E. coli-10	Precision	0.941248	0.941068	0.940913
	Recall	0.849611	0.849611	0.849380
	F1-score	0.893085	0.893004	0.892807
E. coli-20	Precision	0.930314	0.930198	0.930083
	Recall	0.831509	0.831457	0.831367
	F1-score	0.878141	0.878060	0.877959
chr20-10	Precision	0.942144	0.942115	0.942069
	Recall	0.855216	0.855177	0.855110
	F1-score	0.896578	0.896544	0.896486
E. coli	Precision	0.965639	0.964355	0.963755
	Recall	0.918407	0.924131	0.925421
	F1-score	0.941431	0.943815	0.944199
C. elegans	Precision	0.910788	0.897060	0.888170
	Recall	0.949738	0.958047	0.958512
	F1-score	0.929855	0.926551	0.922002
human	Precision	0.767680	0.736254	0.735904
	Recall	0.685903	0.706253	0.706467
	F1-score	0.724491	0.720942	0.720088
